# Web-Based Virtual Learning Environment for Medicine Administration in Pediatrics and Neonatology: Content Evaluation

**DOI:** 10.2196/18258

**Published:** 2020-10-21

**Authors:** Alayne Larissa Martins Pereira, Casandra Genoveva Rosales Martins Ponce Leon, Laiane Medeiros Ribeiro, Guilherme Da Costa Brasil, Karen Karoline Gouveia Carneiro, Géssica Borges Vieira, Yuri Gustavo De Sousa Barbalho, Izabel Cristina Rodrigues Da Silva, Silvana Schwerz Funghetto

**Affiliations:** 1 Universidade de Brasília Brasília Distrito Federal Brazil; 2 Centro Universitário do Distrito Federal Brasília Distrito Federal Brazil; 3 Hospital Universitário Dr. Miguel Riet Corrêa Júnior Rio Grande, Rio Grande do Sul Brazil

**Keywords:** nursing education, health education, educational technology, patient safety.

## Abstract

**Background:**

Worldwide, patient safety has been a widely discussed topic and has currently become one of the greatest challenges for health institutions. This concern is heightened when referring to children.

**Objective:**

The goal of this study was to develop a virtual learning environment for medication administration, as a tool to facilitate the training process of undergraduate nursing students.

**Methods:**

Descriptive research and methodological development with a quantitative and qualitative approach were used with stages of design-based research as methodological strategies. For the development of the virtual environment, 5 themes were selected: rights of medication administration, medication administration steps, medication administration routes, medication calculation, and nonpharmacological actions for pain relief. After development, 2 groups—expert judges in the field of pediatrics and neonatology for environment validation and undergraduate nursing students for the assessment—were used to assess the virtual learning environment. For the validation of the virtual learning environment by expert judges, the content validity index was used, and for the evaluation of the students, the percentage of agreement was calculated.

**Results:**

The study included 13 experts who positively validated the virtual environment with a content validity index of 0.97, and 26 students who considered the content suitable for nursing students, although some adjustments are necessary.

**Conclusions:**

The results show the benefit of the virtual learning environment to the training of nursing students and professional nurses who work in health care. It is an effective educational tool for teaching medication administration in pediatrics and neonatology and converges with the conjectures of active methodologies.

## Introduction

Worldwide, patient safety has been a widely discussed topic, and has currently become one of the greatest challenges for health institutions. It is understood as the decrease, to an acceptable minimum, of the risk of unnecessary harm associated with health care, and although there are recommendations and progress in international literature, scientific knowledge must become incorporated into care practice [1].

The National Patient Safety Program, aligned with the objectives of the World Alliance for Patient Safety of the World Health Organization launched 6 patient safety protocols focusing on the most problematic areas of patient safety, among them improving safety in the prescription, use, and administration of medicines [2].

The concern with patient safety is heightened when referring to children, since they are a vulnerable part of society [3]. The quality of care related to patient safety in nursing is connected to the entire care process, thus, the use of intelligent technologies and the use of standardization can provide greater safety for patients [4].

Communication between professionals, parents, and patients is a factor in pediatric hospitalization, and the consequences of communication failures can generate significant damage to patients and decrease the effectiveness and quality of the care provided. Thus, in pediatric patient care, the prevention of errors, the analysis of the factors that led to an error, aims to implement measures, and improvements contribute to patient safety [5].

It was assumed that the development of a virtual learning environment on medication administration in neonatology and pediatrics would contribute to the knowledge gain of nursing students, and it was postulated that the use of a virtual learning environment is an adequate educational strategy to promote knowledge about pediatric medication administration, encouraging active and motivating learning.

Design-based research involves a new interventionist methodology that seeks to incorporate theoretical aspects of education research with educational practice and offers a promising approach to advance theoretical understanding; although theory alone will not solve all the problems encountered in practice, it can help explain how and why problems can be solved and can help students get better results [6].

Thus, based on the principles of design-based research, to contribute to continued professional education and student learning and to complement the principles of sustainability and patient safety, we developed, validated, and evaluated a virtual learning environment.

## Methods

**Figure 1:**
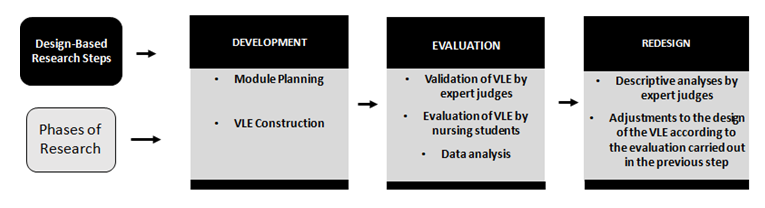
Research phases. VLE: virtual learning environment.

We used descriptive research, methodological development with a quantitative and qualitative approach, and the stages of design-based research as methodological strategies [7]. The phases of the research are described in Figure 1.

For the selection of the themes used in the virtual learning environment, main themes suggested by professionals in a previously conducted study [8] were taken into account, and 5 main themes were then selected: rights of medication administration, medication administration steps, medication administration routes, medication calculation, and nonpharmacological actions for pain relief.

After the construction of the virtual learning environment, 2 groups of participants, with different profiles and purposes, were included: experts in the field of pediatrics and neonatology (teachers and researchers) and final users (undergraduate students of nursing). After construction of the virtual environment, the content was validated by the experts and then evaluated by the students.

Students regularly enrolled in nursing at a public university were considered (convenience sample) and 112 students were invited; 26 students completed the evaluation by answering all the requested instruments corresponding to the final sample.

Data collection with students was carried out from June 2019 to August 2019. Participants were contacted by email and in person. Nursing students, through a questionnaire on Google Forms, filled out a 24-item Likert scale developed in a previous study [9] and adapted for this research. The students judged the virtual environment in terms of interaction and stimulus (6 items); interest and motivation to learn (3 items); dedication, discipline and time management (3 items); communication tools (3 items); didactic material (7 items); and the student's role in the learning process (2 items). Space was reserved for suggestions and additional comments. Quantitative data were calculated using mean and standard deviation using SPSS (version 20; IBM Corp). The percentage of agreement was also calculated, individually and by categories—80% agreement among users was considered as a positive evaluation.

The validation of the virtual learning environment was executed by a committee of specialists with evident knowledge in pediatrics or knowledge of active technologies and with the ability to evaluate the content of the virtual environment. A total of 59 specialists were invited, and 13 completed all the steps and answered all the requested instruments, corresponding to the final sample. To define the specialists, the Fehring Validation Model [10] was used, which consists of criteria used to classify the expertise of professionals in matters covered in educational technology. Nurses with specialized knowledge in pediatrics, neonatology, or active methodologies were included.

The experts answered a structured questionnaire focusing on the nature of the objectives, content, relevance, and environment. In the last section of the questionnaire, a blank space was reserved for the experts to add suggestions and comments (they were able to make qualitative collaborations and contribute to the improvement of the virtual learning environment). The content validity index was used, which measures and evaluates whether the proportion or percentage of experts are in agreement on certain aspects of the instrument and its items (each item individually and then the instrument as a whole). The questionnaire used a Likert-type scale with a score of 1 to 4 [11]. The content validity index of each item was calculated, and the content validity index of each category was calculated. Finally, the mean general content validity index of the virtual learning environment was calculated. For this study, a content validity index of 0.90 was considered as the experts judging the questions to be valid, based on Polit and Beck [12] who establish this value as standard to guarantee the excellence of a scale.

To measure the gain of knowledge, Worral [13] proposed the application of a pretest before the educational intervention, to identify the preacquired knowledge and a posttest to identify the gain of knowledge (retention capacity and memory) as an educational proposal. For the pretest, it was decided to carry out a questionnaire with 13 questions referring to the contents of the virtual environment, and those same questions were asked again in the posttest. The questionnaire contained true or false questions and underwent validation with 3 pediatric specialists, before being made available in the virtual environment. The students' responses to this questionnaire generated data on knowledge gain. With the Wilcoxon statistical test, we compared the outcome of the scores (pretest and posttest).

The research project was approved by the research Ethics Committee of the University of Brasília - Faculdade de Ceilândia, obtaining an opinion favorable to its realization under the protocol no. 3,203,397 and CAAE no. 08880519.3. 0000.8093.

The virtual learning environment for medication administration in pediatrics and neonatology was made available online with free access. The platform used to host the site was Heroku, a cloud computing service, and the reason for choosing this environment was the possibility of inserting images and videos created exclusively for a virtual learning environment.

The environment consisted of approximately 77 pages (HTML language), one of which was the main menu, and 8 were considered submenus of the themes of the modules. After registering, the user received an email, automatically generated by the platform as soon as the administrator approved participation. When accessing the virtual learning environment with login and password, the user was automatically redirected to the pretest page.

**Figure 2:**
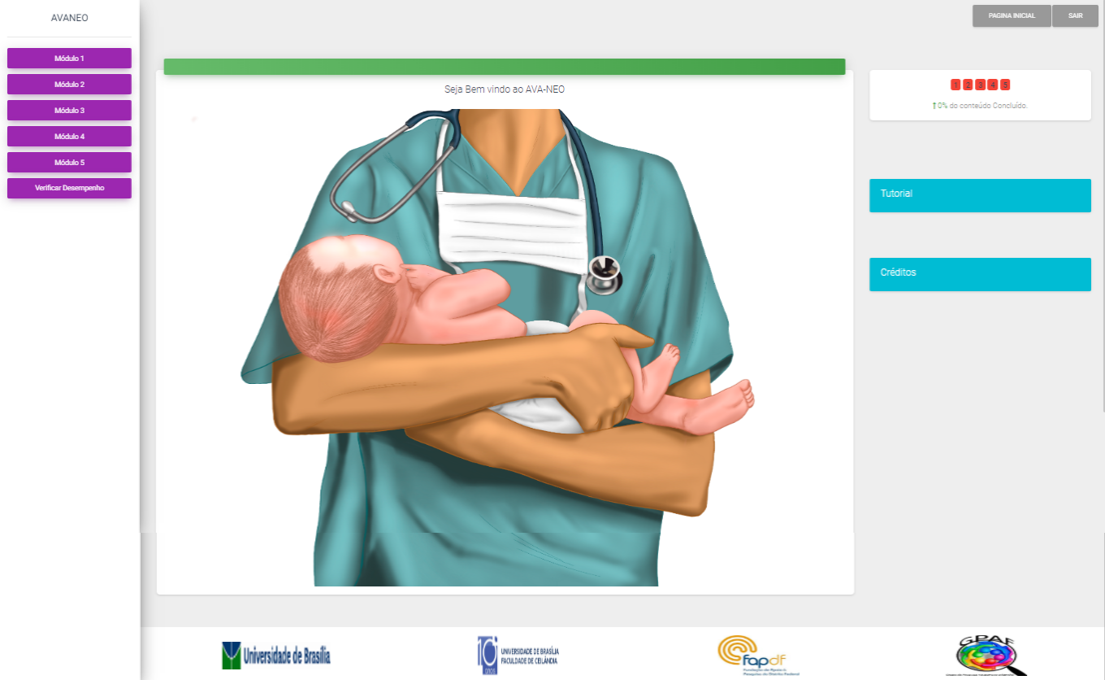
Main menu of the virtual learning environment.

After answering the pretest questions, the user clicked on the *save answers* button and was directed to the main menu (Figure 2). This screen contained a button on the right side that accessed the 5 modules (Table 1), a button to check performance, an indication of the content already viewed, and a button for tutorial and credits. On the access screen for each module, it was possible to observe different interactions. Each circle generated a clickable link that opened a text box. Modules contained complementary material along with the reference used to write the content of that module. The links of the complementary material, when clicked, were directed to the publications.

At the end of the fifth module, the user was automatically redirected to the posttest page to answer the same 13 questions. After saving the answers, the user was directed to the home page and with the check performance button, could review which questions had been correct and which questions had been wrong. For the incorrect questions, a template showing correction and justification appeared.

**Table 1:** Virtual learning environment modules.

Module	Features
Nine rights of medication administration	Right patient, right drug, right route, right time and frequency, right dose, right documentation, right education and information, right pharmaceutical form—each circle opens a text box; complementary materials
Medication administration steps	3 clickable boxes open content on absorption, distribution, and elimination, complementary materials
Medication administration routes	4 videos
Medication calculation	Formulas for calculating medication doses, complementary materials
Nonpharmacological actions for pain relief	Images with accompanying text, complementary materials

## Results

### Validation

The sample was composed of 13 nursing professors; 69% (9/13) had greater than 10 years of professional experience, and 46% (6/13) had a doctorate, and 46% (6/13) has a master's degree.

The experts’ ages ranged from 25 to 55 years, and 31% (8/13) of the sample were aged 25 to 35 years; 85% (11/13) of the experts were female. Regarding having experience in the area of active teaching methodologies, 100% (13/13) answered “yes.” Of the 13 experts, 54% (7/13) had publications in the area of pediatrics and neonatology.

Table 2 describes the assessments of the criteria analyzed and judged by the expert judges. No judge rated the analysis criteria as “Inadequate.” The content validity index by category ranged from 0.91 to 1.0. The total content validity index reached was 0.97, which successfully validated the content.

**Table 2:** Responses from experts and content validity index results.

Items		Inadequate, n	A little adequate, n	Quite adequate, n	Very adequate, n	CVI^a^
**Nature of the objectives**						
	Was the purpose of the VLE^b^ clear? (described in the Tutorial tab of the platform)	0	0	3	10	1.0
	Are the verbs chosen accurately in terms of expected behavior?	0	0	5	8	1.0
	Are the objectives consistent with the content presented?	0	0	3	10	1.0
**Content**						
	Does the content meet the theme and objectives proposed?	0	1	5	7	0.92
	Is the content updated and does it contain correct information (sources and references)?	0	2	1	10	0.85
	Are the texts easy to read?	0	1	2	10	0.92
	Is the writing style compatible with the level of knowledge of the academics?	0	1	1	11	0.92
	Is the number of modules sufficient (adequate division)?	0	1	6	6	0.92
	Is the sequence of the modules adequate (clear and well-structured information)?	0	1	4	8	0.92
**Relevance**						
	Do the items cover important aspects of nursing practice in the Administration of Medicines in Neonatology?	0	0	2	11	1.0
	Does (the) VLE contribute to learning and knowledge acquisition?	0	0	3	10	1.0
**Ambiance**						
	Is the VLE suitable for presenting the content?	0	0	1	12	1.0
	Does VLE encourage student autonomy?	0	1	3	9	0.92
	Do VLE resources offer learning situations?	0	0	4	9	1.0

^a^CVI: content validity index.

^b^VLE: virtual learning environment.

### Evaluation

Of the 26 students who participated in the evaluation, 81% (21/26) were aged 19 to 24 years, 62% (16/26) are female and 38% (10/26) are male. The majority of students (17/26, 65%) were in the seventh semester (of 10) in undergraduate nursing.

Of the 24 items that were analyzed, 22 items and 5 of the 6 categories reached the stipulated value of 80% concerning percentage of agreement (Table 3). The categories *interaction and stimulus*, *instructional material*, and *the student's role in the learning process* had the best percentage of agreement values (96% agreement). The category *communication tools* obtained 78% percent agreement, which was close to the stipulated value, indicating the need for improvements regarding the exchange of information between users and the administrator of the virtual environment. The mean Cronbach α was 0.88, indicating a high degree of reliability and consistency; users considered the content of the virtual learning environment suitable for nursing students, although some adjustments are necessary, for example, communication tools, which obtained the lowest percentage of agreement.

**Table 3:** Criteria evaluated by the users (n=26).

Item		Rating, mean (SD)	Agreement, %
**Interaction and stimulation**		**4.70 (0.47)**	**96**
	The VLE^a^ is easy to interact with and arouses your interest in the subject	4.73 (0.45)	100
	The environment enables learning situations	4.69 (0.62)	92
	VLE allows you to freely browse through content	4.69 (0.68)	96
	The activities are relevant and meet the objectives proposed	4.77 (0.43)	100
	Access to modules is easy	4.73 (0.53)	96
	The VLE instigates/invites change in behavior and attitude	4.62 (0.64)	92
**Interest and motivation to learn**		**4** **.59 (0.62)**	**93**
	The VLE NEO-PED^b^ motivates the students to learn about medication administration in neonatology and pediatrics	4.81 (0.40)	100
	The VLE presents complementary readings and notes on the course content	4.50 (0.76)	92
	The VLE encourages the desire to study beyond the content presented	4.46 (0.71)	88
**Dedication, discipline, and time management**		**4** **.55 (0.66)**	**92**
	During the access to the VLE, time was organized for online course activities	4.38 (0.80)	88
	During access to the VLE, there was self-discipline for online teaching	4.62 (0.64)	92
	During the access to the VLE, there was access to the modules with the regularity proposed	4.65 (0.56)	96
**Communication tools**		**4** **.22 (1.04)**	**78**
	The environment encourages the exchange of information with colleagues	4.19 (1.10)	77
	The links provided are relevant to the learning content	4.62 (0.64)	92
	An e-mail was used to answer questions about the content	3.85 (1.38)	65
**Courseware**		**4** **.71 (0.56)**	**96**
	The VLE is explanatory and easy to understand	4.69 (0.55)	92
	The content is well divided between the modules	4.69 (0.55)	96
	Information is presented in a logical and coherent way	4.65 (0.56)	96
	The writing style is easy to understand	4.69 (0.55)	96
	The colors and size of the letters are adequate	4.73 (0.67)	96
	The images contribute to learning	4.85 (0.37)	100
	The media are correlated with the content and provide a complement to the texts	4.73 (0.67)	96
**Role of the student in the learning process**		**4** **.63 (0.55)**	**96**
	You consider having freedom in building your knowledge	4.54 (0.58)	96
	You believe you are responsible for your learning process	4.73 (0.53)	96

^a^VLE: virtual learning environment.

^b^NEO-PED: neonatology and pediatrics.

The results of the comparison between pretest and posttest correct answers are shown in the table below (Table 4). The pre–post difference in number of correct answers was statistically significant (*P*=.02).

**Figure 3:**
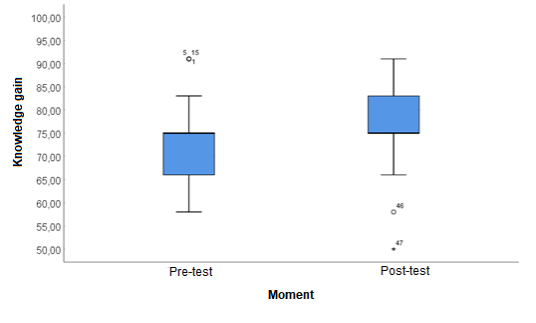
Knowledge gain before and after use.

Figure 3 likewise demonstrates the significant increase, corroborating the purpose of this study, and that students obtained a considerable gain of knowledge.

**Table 4:** Comparison of knowledge gain before and after use of the virtual environment.

Knowledge gain	Score, median (IQR)	*P* value
Pretest	75 (66-75)	.02
Posttest	75 (75-83)	

For the last stage, according to design-based research, there is a refinement of the intervention or re-design, in which adjustments are made to the design of the virtual learning environment according to the assessment that was made in the previous stage. At the end of the instrument, the expert judges had the opportunity to provide suggestions, changes, or opinions on the virtual environment. These opinions served as the basis for the changes in the virtual learning environment as a whole and not just in the content. Most of the comments emphasized the importance of virtual learning environment and its quality and excellence in this production. The suggestions were accepted, and changes were made as proposed by the experts.

## Discussion

### General

Through a bibliographic survey on the topic of medication administration in pediatrics and neonatology, it was possible to perceive the scarcity of research related to the use of virtual learning environments focused on this theme. Our objective was to develop a virtual learning environment with the theme of medication administration, as a facilitating tool for the training of undergraduate nursing students.

To refine the intervention of this study, descriptive analyses of the expert judges' opinions were used, as suggested at the end of the questionnaire. It should be noted that the judges' suggestions were analyzed, and the contents were modified, taking into consideration the target audience of this study.

According to the literature, electronic materials that provide information that contributes to the construction of knowledge are resources that support learning, especially for young students [14].

Virtual learning environments have been placed as innovative strategies that provide paradigm shifts for health care professionals; their development and use provide new educational possibilities to be explored both in universities and in continuing education [15]. Developing care techniques in safe environments for patients and professionals has become a priority for health systems, mainly in the development of strategies to prevent failures that affect good hospital practices [16]. By providing digital education, virtual environments have the potential to promote health education to the nursing team and are thus being used as an alternative or complement to traditional education for health professionals [17].

Measuring knowledge gained with the virtual environment showed an increase in correct answers in the posttest in comparison to the number of correct answers in the pretest. This finding proves that the online learning strategy can be an educational teaching resource to be used in the training of nurses or as a permanent education resource.

The use of educational technologies makes important contributions to the nursing teaching process, as they favor the enrichment of the academic's cognitive structure by enabling continuous access to information, for significant learning, in which the acquired knowledge is stored and can be remembered for a longer time [18].

The potential of virtual learning environments is related to the use of several elements simultaneously from mechanisms of interaction and interactivity that support the purpose of their construction, and in nursing, the use of virtual learning environments does not replace the organic relationships between subjects, the use of this technology must occur concurrently with other teaching methodologies [19].

As a strategy used to streamline and stimulate users in the navigation of virtual learning environment, videos were included, as technological tools, and by combining elements such as images, texts, and sounds in a single object, we sought to motivate and engage users in the teaching-learning process to guarantee the promotion of knowledge [20-24].

The students positively highlighted their participation in the process of building educational technologies, as well as their experience and autonomy when faced with an innovative object in their training.

For resources such as virtual environments to favor the construction of knowledge and to demonstrate knowledge gains in these educational settings, it is necessary to promote the student's interest, adapt to their needs, and adapt to their cognitive style, thus allowing a more dynamic and active learning process [25].

### Conclusion

The educational technology was validated by experts, as well as being positively evaluated by students. The knowledge acquired by the students from the virtual learning environment was confirmed by statistical analyses that demonstrate the potential of this tool as an active teaching methodology to enrich and complement the traditional teaching method. The results prove the virtual learning environment to be an effective educational tool for teaching medication administration in pediatrics and neonatology and converge with the assumptions of active methodologies. Thus, the virtual learning environment of this study was established as a complementary didactic resource for theoretical teaching at the undergraduate level and the permanent education of nursing professionals.

No studies were found using design-based research in nursing with the creation of virtual learning environments, however, studies in the areas of biology, physics, medicine and psychology, education were found, which can be explained as it is a methodology that is considered to be new, although the results obtained validate the necessary efforts since the knowledge and the didactic products acquired are linked to the natural environment from which the initial problems arose and which motivated the research [26].

For future research, larger samples are suggested, for more detailed statistical power, in addition to the creation of new modules to cover some new suggested topics and the development of more videos based on the necessary demand.
